# Association between adiposity and psoriatic arthritis beyond classical clinical predictors

**DOI:** 10.1007/s10067-026-08353-y

**Published:** 2026-08-11

**Authors:** Estefanía Pardo, Stefanie Burger, Marta Loredo, Ignacio Braña, Paula Alvarez, Rubén Queiro

**Affiliations:** 1 Rheumatology Division, Hospital Universitario Central de Asturias, Oviedo, Spain; 2 Department of Medicine, Faculty of Medicine and Health Sciences, Oviedo University, Oviedo, Spain; 3 Rheumatology and ISPA Translational Immunology Division, Hospital Universitario Central de Asturias, Avenida de Roma, S/N 33011 Oviedo, Spain

**Keywords:** Cardiometabolic burden, Disease risk, Psoriasis, Psoriatic arthritis, Waist circumference

## Abstract

**Background:**

Psoriatic arthritis (PsA) develops in a substantial proportion of patients with psoriasis. Although obesity has been linked to PsA, whether adiposity independently amplifies articular risk beyond established predictors remains incompletely defined.

**Objective:**

To explore whether adiposity showed a graded association with PsA across different clinical strata.

**Methods:**

This cross-sectional study included 519 psoriatic disease cases (27.2% with PsA). Multivariable logistic regression models assessed the association of waist circumference (per 10 cm) and body mass index (BMI) with PsA, adjusting for psoriasis duration ≥ 10 years, nail involvement, PASI, and sex. Interaction terms between adiposity measures and classical predictors were explored. Model discrimination was evaluated using the area under the receiver operating characteristic curve (AUC), and correlated AUCs were compared using the DeLong test.

**Results:**

Nail involvement (OR 3.31, *p* < 0.001) and psoriasis duration (OR 2.70, *p* < 0.001) were the strongest independent associations with PsA. An ordinal quartile-based model supported a graded association across waist circumference categories (aOR per quartile increase 1.28, 95% CI 1.05–1.56; *p* = 0.016). BMI showed a comparable association, and discrimination was similar between adiposity measures (AUC 0.74 vs 0.73; DeLong *p* = 0.33). No significant interaction was observed between adiposity and psoriasis duration or severity. Exploratory interaction analyses suggested that the adiposity–PsA association may differ according to nail involvement status.

**Conclusions:**

Adiposity was independently associated with the presence of PsA and showed a graded relationship across adiposity levels. These findings support a potential link between metabolic burden and articular disease expression in psoriatic disease.

**Supplementary Information:**

The online version contains supplementary material available at https://doi.org/10.1007/s10067-026-08353-y.

**Table:** 

Key points • *Adiposity was independently associated with psoriatic arthritis beyond classical clinical predictors*. • *Waist circumference and BMI showed comparable graded associations with psoriatic arthritis probability*. • *The adiposity–psoriatic arthritis association appeared stronger in patients with nail involvement*.	

## Introduction

Psoriatic arthritis (PsA) develops in up to 30% of patients with psoriasis and represents a major contributor to long-term disability. Several clinical predictors of transition from cutaneous to articular disease have been consistently identified, including nail involvement, long-standing psoriasis, and greater skin severity. These factors suggest that cumulative inflammatory burden and specific phenotypic features may predispose to articular involvement [1, 2].

Obesity has also been associated with PsA in epidemiological studies, yet its precise role remains debated. Most available evidence relies on body mass index (BMI), a measure of overall adiposity that does not distinguish between peripheral and central fat distribution. Given the recognized link between central adiposity, systemic inflammation, and cardiometabolic risk, abdominal fat accumulation may represent a biologically relevant contributor to articular disease development beyond classical predictors [3, 4].

Whether adiposity independently amplifies PsA risk after accounting for established clinical factors remains insufficiently explored. In particular, the relative contribution of waist circumference compared with BMI in this context has not been clearly defined.

The aim of the present study was therefore to evaluate the independent association between adiposity measures and PsA in patients with psoriatic disease, adjusting for classical predictors, and to explore whether adiposity is independently associated with PsA beyond established clinical predictors.

## Methods

### Study design and population

We conducted a cross-sectional analysis of consecutive adult patients with psoriatic disease evaluated at a tertiary care center. Patients with complete data on anthropometric and clinical variables were included. Psoriatic arthritis (PsA) was defined as physician-confirmed inflammatory arthritis fulfilling the Classification Criteria for Psoriatic Arthritis (CASPAR). The study was conducted in accordance with the Declaration of Helsinki and approved by the ethics committee of the Principality of Asturias, Spain (#CEImPA 2025.102). All patients provided informed consent for the use of anonymized clinical data.

### Clinical and anthropometric variables

Demographic data, psoriasis duration, nail involvement, and Psoriasis Area and Severity Index (PASI) were recorded. Long-standing psoriasis was defined as disease duration ≥ 10 years. High skin activity was defined as PASI ≥ 10. Adiposity was assessed using waist circumference (cm) and body mass index (BMI, kg/m^2^). Waist circumference was analyzed as a continuous variable (per 10 cm increase). BMI was analyzed as a continuous variable and, in sensitivity analyses, as an alternative adiposity measure. Body weight was also recorded and included in descriptive analyses. In addition, waist circumference was categorized into quartiles to assess whether the association with PsA followed a graded pattern across clinically interpretable adiposity strata.

### Statistical analysis

Associations between adiposity measures and PsA were evaluated using multivariable logistic regression models adjusted for established predictors: psoriasis duration ≥ 10 years, nail involvement, PASI ≥ 10, and sex. Age was evaluated but excluded from the final parsimonious model due to lack of independent association after adjustment. Model discrimination was assessed using the area under the receiver operating characteristic curve (AUC). Correlated AUCs between waist circumference– and BMI-based models were compared using the DeLong test. Predicted probability curves were generated to illustrate the graded relationship between adiposity measures and PsA risk, stratified by nail involvement and psoriasis duration, with sex-specific analyses performed. Interaction terms between adiposity measures and classical predictors (nail involvement, psoriasis duration, and PASI) were explored in extended models. To assess the incremental contribution of adiposity, changes in McFadden pseudo-R^2^ and AUC were evaluated between the base model (classical predictors only) and the extended model including waist circumference and BMI. Illustrative predicted probabilities of PsA were calculated for representative clinical profiles. Statistical significance was defined as *p* < 0.05.

## Results

Of 572 patients with available PsA status, 519 had complete data for variables included in the multivariable model and were analyzed. Among the 519 cases, PsA was present in 141 patients (27.2%). Patients with PsA had a longer duration of psoriasis and more frequent nail involvement compared with those without articular disease. Measures of adiposity were also higher among PsA patients (Table 1). Baseline characteristics of the full cohort (*n* = 572) are provided in Supplementary Table S1.

**Table 1:** Baseline characteristics of patients with psoriatic disease according to the presence of psoriatic arthritis

Variable	No PsA (*n* = 378)	PsA (*n* = 141)	*p*-value
Female sex, n (%)	165 (43.7%)	80 (56.7%)	0.011
Psoriasis duration, years	17.3 (14.6)	23.1 (14.5)	< 0.001
Psoriasis duration ≥ 10y, n (%)	224 (59.3%)	116 (82.3%)	< 0.001
PASI	11.2 (11.7)	13.7 (12.0)	0.036
PASI ≥ 10, n (%)	167 (44.2%)	86 (61.0%)	0.001
Nail involvement, n (%)	196 (51.9%)	113 (80.1%)	< 0.001
Waist circumference, cm	95.8 (14.3)	98.9 (13.3)	0.024
BMI, kg/m^2^	27.2 (4.8)	28.4 (4.9)	0.017
Body weight, kg	77.3 (16.4)	78.2 (15.1)	0.578

Values are mean (SD) or n (%). Group comparisons were performed using Student’s t-test or chi-square test, as appropriate. PsA was defined according to CASPAR criteria

In parsimonious multivariable models adjusted for sex, psoriasis duration ≥ 10 years, nail involvement, and PASI ≥ 10, nail involvement emerged as the strongest independent association with PsA (OR 3.31, 95% CI 2.02–5.42), followed by psoriasis duration ≥ 10 years (OR 2.70, 95% CI 1.60–4.56). Waist circumference was independently associated with PsA, with each 10 cm increase conferring a 32% increase in odds (OR 1.32, 95% CI 1.12–1.55), whereas PASI ≥ 10 was not independently associated after adjustment (OR 1.42, 95% CI 0.91–2.20). Substitution of waist circumference with BMI yielded comparable results, with BMI (per 5 kg/m^2^) independently associated with PsA (OR 1.37, 95% CI 1.10–1.71) and similar overall discrimination (AUC 0.74 vs 0.73, DeLong *p* = 0.33), supporting the robustness of the adiposity–risk association irrespective of the anthropometric index used. In an exploratory quartile-based analysis, PsA prevalence increased from 22.6% in the lowest waist circumference quartile to 30.1% in the highest quartile. After adjustment for sex, psoriasis duration ≥ 10 years, nail involvement and PASI ≥ 10, the third and fourth quartiles were associated with approximately two-fold higher odds of PsA compared with the lowest quartile. An ordinal quartile-based model supported a graded association across waist circumference categories (aOR per quartile increase 1.28, 95% CI 1.05–1.56; *p* = 0.016) (Supplementary Table S2). Including both adiposity measures in the same model did not meaningfully improve discrimination. Compared with the base model including classical predictors only, addition of waist circumference modestly improved model discrimination (AUC 0.72 to 0.74) and increased McFadden pseudo-R^2^ from 0.11 to 0.13. A similar but slightly smaller improvement was observed when BMI was added to the base model (AUC 0.72 to 0.73; pseudo-R^2^ 0.11 to 0.12). Although statistically measurable, the incremental improvement in discrimination was modest and should not be interpreted as establishing clinical predictive utility. Table 2 depicts both regression models. Illustrative predicted probabilities derived from the parsimonious waist model are shown in Supplementary Table S3.

**Table 2:** Multivariable logistic regression models for psoriatic arthritis

Model 1: Waist circumference (per 10 cm)			
Variable	OR	95% CI	*p*-value
Male sex	0.36	0.22–0.56	< 0.001
Waist circumference (per-10 cm)	1.32	1.12–1.55	< 0.001
Psoriasis duration ≥ 10 years	2.70	1.60–4.56	< 0.001
Nail involvement	3.31	2.02–5.42	< 0.001
PASI ≥ 10	1.42	0.91–2.20	0.12
AUC = 0.74. McFadden pseudo-R^2^ = 0.13			
Model 2: Body mass index (per 5 kg/m^2^)			
Variable	OR	95% CI	*p*-value
Male sex	0.42	0.27–0.65	< 0.001
BMI (per-5 kg/m^2^)	1.37	1.10–1.71	0.005
Psoriasis duration ≥ 10 years	2.58	1.54–4.34	< 0.001
Nail involvement	3.36	2.06–5.49	< 0.001
PASI ≥ 10	1.38	0.90–2.12	0.14
AUC = 0.73. McFadden pseudo-R^2^ = 0.12			

Multivariable logistic regression models assessing the association between adiposity measures and psoriatic arthritis. Model 1 includes waist circumference (per 10 cm increase), and Model 2 includes BMI (per 5 kg/m^2^ increase). Both models are adjusted for psoriasis duration ≥ 10 years, nail involvement, PASI ≥ 10, and sex. OR, odds ratio; CI, confidence interval; AUC, area under the receiver operating characteristic curve

Predicted probability curves demonstrated a progressive increase in PsA risk across the distribution of adiposity measures. This graded pattern was consistent across strata defined by nail involvement and disease duration, and similar risk gradients were observed in sex-stratified analyses, although baseline probabilities were lower in men (Supplementary Figure S1).

**Fig. 1:**
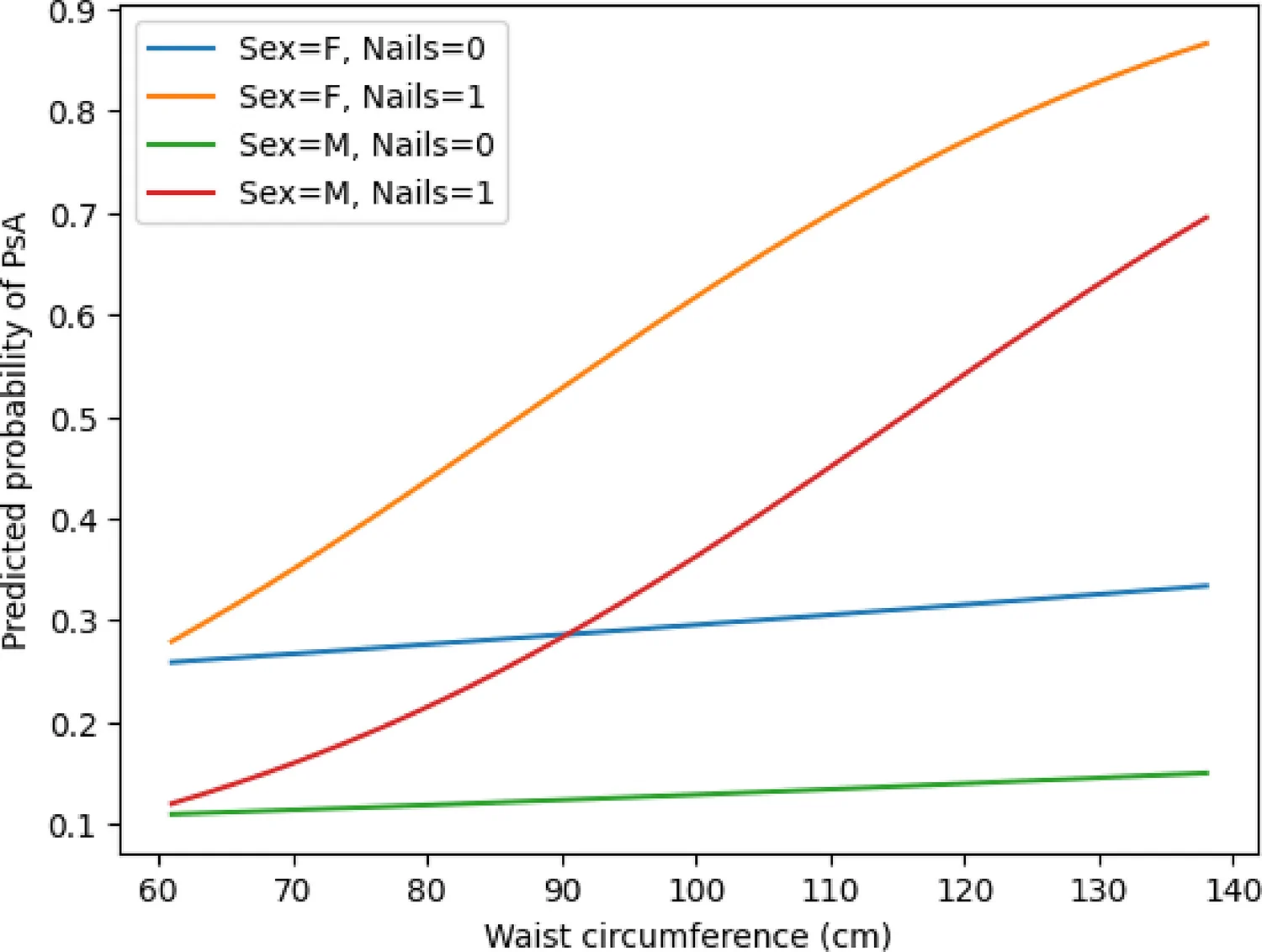
Predicted probability of psoriatic arthritis according to waist circumference, stratified by sex and nail involvement. Curves were derived from a multivariable logistic regression model including waist circumference (per 10 cm), nail involvement, psoriasis duration ≥ 10 years, PASI ≥ 10, and sex, including exploratory interaction analyses according to nail involvement status. Predicted probabilities are shown for patients with PASI ≥ 10 and disease duration ≥ 10 years. F: female; M: male

In interaction analyses, no significant interaction was observed between waist circumference and psoriasis duration ≥ 10 years (*p* = 0.51). An exploratory interaction pattern was identified between waist circumference and nail involvement (interaction term OR 1.38, 95% CI 0.99–1.92, *p* = 0.06). Stratified predicted probability curves suggested a steeper adiposity–risk gradient among patients with nail involvement compared with those without. In patients without nail disease, the increase in PsA probability across waist circumference values was modest, whereas in those with nail involvement, the risk gradient appeared substantially amplified, particularly at higher adiposity levels (Fig. 1).

When psoriasis severity was incorporated as a continuous variable in extended interaction models, no significant three-way interaction between waist circumference, nail involvement, and PASI was observed (*p* = 0.32), and no consistent interaction between waist circumference and PASI alone was identified. The interaction between waist circumference and nail involvement remained borderline (OR 1.58, 95% CI 0.93–2.69, *p* = 0.089), suggesting that any potential metabolic amplification effect may be more closely linked to structural susceptibility than to overall skin severity.

## Discussion

In this cross-sectional analysis of patients with psoriatic disease, adiposity showed an independent and graded association with the presence of PsA. Nail involvement and long-standing psoriasis remained the strongest determinants of articular disease, consistent with previous literature. However, both waist circumference and BMI retained independent associations after adjustment, and predicted probability curves demonstrated a progressive increase in PsA likelihood across adiposity levels. These findings support the concept that metabolic burden may be linked to phenotypic expression of psoriatic disease rather than representing a simple comorbidity.

The association between obesity and PsA has been reported in epidemiological studies, including population-based cohorts suggesting that higher BMI increases the risk of developing PsA among patients with psoriasis [3, 5–8]. Nevertheless, most prior studies have relied on BMI as a categorical exposure and have not systematically adjusted for key phenotypic predictors such as nail involvement or disease duration. In addition, the incremental contribution of central adiposity beyond overall adiposity has been insufficiently explored. Our analysis extends this evidence by demonstrating that adiposity retains an independent association even after accounting for established clinical predictors, and that the relationship appears graded rather than dichotomous.

The graded risk pattern observed in predicted probability curves is particularly informative. Rather than identifying a specific “threshold” at which adiposity becomes relevant, the data suggest a progressive increase in PsA risk across the adiposity continuum. This argues against a purely binary interpretation of obesity in psoriatic disease and instead supports a model in which increasing metabolic burden may contribute to a permissive inflammatory environment associated with articular disease expression. Importantly, this pattern was consistent across strata defined by nail involvement and disease duration, indicating that adiposity does not replace classical predictors but may amplify their impact.

An additional observation further supports this interpretation. Although formal statistical interaction between waist circumference and nail involvement did not reach conventional significance, predicted probability curves suggested a steeper adiposity–risk gradient among patients with nail disease. In contrast, no consistent interaction was observed between adiposity and psoriasis severity, and extended models did not support a three-way interaction between waist circumference, nail involvement, and skin severity.

These findings suggest that the potential metabolic amplification effect may be more closely linked to structural susceptibility than to overall skin severity. Nail involvement has long been considered a marker of enthesial–articular predisposition within the psoriatic disease spectrum [9–11]. The apparent strengthening of the adiposity–risk gradient in this subgroup may be compatible with a conceptual framework in which metabolic and structural factors coexist in patients with higher PsA probability [1, 2].

Although causal inference cannot be established in a cross-sectional study, from a mechanistic perspective, these findings are biologically plausible. Adipose tissue, particularly visceral fat, is an active endocrine organ that produces pro-inflammatory cytokines and adipokines, including TNF, IL-6, leptin, and resistin [3, 4, 12]. These mediators contribute to systemic low-grade inflammation and may enhance Th17-driven immune responses, a central pathway in both psoriasis and PsA. Chronic metabolic inflammation could therefore create a permissive immunological environment that facilitates synovial inflammation once additional triggers are present. In this framework, adiposity may function as a modulator of inflammatory set point rather than as a primary cause of articular disease. In addition, mechanical stress at entheses may represent an additional pathway linking adiposity and PsA, beyond systemic metabolic inflammation [3].

The comparison between waist circumference and BMI provides additional insight. Model discrimination was similar between adiposity measures, and correlated ROC curves did not differ significantly. This suggests that the metabolic signal observed is robust across anthropometric indices. Rather than indicating superiority of one metric over another, these findings reinforce the interpretation that overall adiposity burden—regardless of measurement approach—contributes to articular risk. Additionally, body weight did not differ significantly between patients with and without PsA, whereas waist circumference and BMI did. This supports the interpretation that the observed association is not simply explained by total body mass but may reflect adiposity distribution and/or body composition-related metabolic burden.

Sex-stratified analyses further support the consistency of this relationship. Although baseline probabilities differed between men and women, the slope of the adiposity–risk gradient was similar across sexes, arguing against a purely sex-driven confounding effect. The metabolic contribution to articular risk therefore appears to operate independently of sex-specific baseline differences in PsA prevalence.

Several limitations should be acknowledged. The cross-sectional design precludes temporal or causal inference. Reverse causation cannot be excluded, as patients with established PsA may develop increased adiposity secondary to reduced physical activity, functional limitation, or treatment-related factors. Approximately 9–10% of patients with available PsA status were excluded from the multivariable analyses due to incomplete data. Although baseline patterns in the full cohort were broadly similar, complete-case analysis may have introduced selection bias or influenced effect estimates. However, baseline patterns in the full cohort were consistent with the analytic sample. Residual confounding cannot be excluded, although models were adjusted for established clinical predictors. In addition, the single tertiary-care setting may be enriched by patients with more severe or complex psoriatic disease, potentially limiting generalizability.

Despite these limitations, this study has notable strengths. Psoriatic arthritis diagnosis was based on CASPAR criteria, ensuring standardized case definition. The sample size permitted multivariable modeling with adequate statistical power, and the consistency of findings across adiposity measures and sex-stratified analyses enhances internal validity. The use of predicted probability curves provides clinically interpretable visualization of the risk gradient, moving beyond categorical interpretations of obesity. The illustrative probabilities provided in Supplementary Table S2 further highlight the cumulative and amplifying effect of clinical susceptibility markers and adiposity on PsA risk. However, these estimates should be interpreted as exploratory and require external validation before clinical application.

In conclusion, adiposity appears to independently amplify articular risk in psoriatic disease beyond classical predictors such as nail involvement and long-standing psoriasis. The graded and sex-consistent nature of this association supports a model of metabolic–inflammatory synergy in PsA pathogenesis. Rather than viewing obesity solely as a comorbidity, these findings suggest that metabolic burden may modulate the threshold for articular disease within a multifactorial pathogenic framework. Prospective studies are warranted to clarify whether modifying metabolic status can influence the natural history of psoriatic arthritis.

## Supplementary Information

Below is the link to the electronic supplementary material.

Supplementary file1 (DOCX 129 KB)

## Data Availability

All data included in the present study are available to third parties under reasonable request.
