# Supplementary material for: Association between adiposity and psoriatic arthritis beyond classical clinical predictors
Source: Clin Rheumatol. 2026 Aug 11;45(10):6153–8. doi: 10.1007/s10067-026-08353-y (PMC13582208; doi:10.1007/s10067-026-08353-y)
Supplement: Supplementary file 1 [file 10067_2026_8353_MOESM1_ESM.docx]

**Supplementary material. Association between Adiposity and Psoriatic Arthritis Beyond Classical Clinical Predictors**

**Table S1. Baseline disease features of the study cohort (n: 572)**

| **Variables** | **Total**  **n: 572** | **Men**  **n: 309** | **Women**  **n: 263** |
| --- | --- | --- | --- |
| Age (yrs), mean (SD) | 46.7 (14.5) | 47.5 (14.6) | 45.7 (14.3) |
| Age at disease onset (yrs), median (min, max) | 23.0 [1.0, 78.0] | 24.5 [1.0, 74.0] | 20.0 [1.0, 78.0] |
| Education level: |  |  |  |
| Primary, n (%) | 163 (28.5) | 111 (35.9) | 52 (19.8) |
| Secondary, n (%) | 262 (45.8) | 130 (42.1) | 132 (50.2) |
| University, n (%) | 147 (25.7) | 68 (22.0) | 79 (30.0) |
| Duration (yrs), mean (SD) | 19.4 (14.8) | 18.7 (14.3) | 20.3 (15.4) |
| PsA, n (%) | 171 (30) | 81 (26.2) | 90 (34.2) |
| Weight, mean (SD) | 77.9 (16.4) | 85.4 (14.6) | 69.3 (13.9) |
| BMI, mean (SD) | 27.6 (5.02) | 28.5 (4.43) | 26.7 (5.48) |
| Waist perimeter (cm), mean (SD) | 96.7 (14.1) | 101 (11.6) | 91.4 (14.7) |
| PASI, mean (SD) | 15.1 (12.0) | 16.0 (11.9) | 14.0 (12.0) |
| PASI ≥ 10, n (%) | 294 (51.4%) | 174 (56.3) | 120 (45.6%) |
| Nail disease, n (%) | 328 (57.3%) | 192 (62.1) | 136 (51.7%) |
| Plaque psoriasis, n (%) | 497 (86.9) | 281 (90.9) | 216 (82.1) |
| Smoking, n (%) | 196 (34.3) | 99 (32.0) | 97 (36.9) |
| Alcohol consumption (SDU), median (min, max) | 0 [0.0, 30.0] | 0 [0.0, 2.0] | 0 [0.0, 30.0] |
| T1D, n (%) | 22 (3.8) | 11 (3.6) | 11 (4.2) |
| T2D, n (%) | 45 (7.9) | 29 (9.4) | 16 (6.1) |
| Hypertension, n (%) | 114 (20.0) | 68 (22.0) | 46 (17.5) |
| Dyslipidemia, n (%) | 113 (19.8) | 68 (22.0) | 45 (17.1) |
| NAFLD, n (%) | 129 (22.6) | 99 (32.0) | 30 (11.4) |
| Patients with CHD, n (%) | 33 (5.8) | 20 (6.5) | 13 (4.9) |
| Patients on systemic therapy, n (%) | 370 (64.7) | 203 (65.7) | 167 (63.5) |
| Biologics, n (%) | 233 (40.7) | 125 (40.5) | 108 (41.1) |
| Anti-TNF | 118 (20.6) | 63 (20.4) | 55 (20.9) |
| Anti IL12/23 | 63 (11.0) | 35 (11.3) | 28 (10.6) |
| Anti-IL17 | 52 (9.1) | 27 (8.8) | 25 (9.5) |

yrs: years; PsA: psoriatic arthritis; BMI: body mass index; PASI: psoriasis area and severity index; SDU: standard drink unit; T1D: type 1 diabetes; T2D: type 2 diabetes; NAFLD: non-alcoholic fatty liver disease; CHD: coronary heart disease; TNF: tumor necrosis factor; IL: interleukin.

**Figure S1. Predicted probability of psoriatic arthritis according to waist circumference in men and women with PASI ≥10**

**Men**

**Women**

Predicted probability of psoriatic arthritis (PsA) across increasing waist circumference values in men with PASI ≥10, derived from the final parsimonious multivariable logistic regression model. Curves are stratified according to nail involvement (NAILS=0/1) and psoriasis duration ≥10 years (DUR10=0/1). The model was adjusted for waist circumference (per 10 cm), nail involvement, psoriasis duration ≥10 years, PASI ≥10, and sex. The figure illustrates a progressive increase in PsA probability with increasing adiposity, with a steeper gradient observed in patients with nail involvement and long-standing psoriasis.

**Supplementary Table S2. Prevalence of psoriatic arthritis according to waist circumference quartiles**

| Waist circumference quartile | Waist circumference range (cm) | n | PsA, n (%) |
| --- | --- | --- | --- |
| Q1 | 61–88 | 137 | 31 (22.6%) |
| Q2 | 89–98 | 137 | 38 (27.7%) |
| Q3 | 99–106 | 122 | 36 (29.5%) |
| Q4 | 107–138 | 123 | 37 (30.1%) |

Values are presented as number (%) unless otherwise indicated. PsA: psoriatic arthritis.

**Adjusted association between waist circumference quartiles and psoriatic arthritis**

| **Waist circumference quartile** | **Adjusted OR** | **95% CI** | **p-value** |
| --- | --- | --- | --- |
| Q1 (reference) | 1.00 | — | — |
| Q2 | 1.54 | 0.85–2.79 | 0.159 |
| Q3 | 2.02 | 1.08–3.78 | 0.027 |
| Q4 | 2.10 | 1.11–3.97 | 0.022 |

Ordinal quartile trend analysis: adjusted OR per quartile increase = 1.28 (95% CI 1.05–1.56), p=0.016. Multivariable logistic regression adjusted for sex, psoriasis duration ≥10 years, nail involvement, and PASI ≥10. OR: odds ratio; CI: confidence interval.

**Table S3. Illustrative predicted probabilities of PsA derived from the parsimonious waist circumference model**

Values represent predicted probability (%) of PsA according to clinical profile.

Female. Waist circumference = 90 cm

| **Clinical scenario** | **Predicted probability (%)** |
| --- | --- |
| Low clinical risk (no nails, <10y, PASI<10) | 8.5 |
| PASI ≥10 only | 12.4 |
| Duration ≥10 years only | 20.1 |
| Nails only | 27.8 |
| Duration + PASI ≥10 | 26.2 |
| Nails + PASI ≥10 | 39.3 |
| Nails + Duration | 45.4 |
| High clinical risk (nails + ≥10y + PASI≥10) | 54.1 |

Female. Waist circumference = 100 cm

| **Clinical scenario** | **Predicted probability (%)** |
| --- | --- |
| Low clinical risk | 11.3 |
| PASI ≥10 only | 16.1 |
| Duration ≥10 years only | 25.7 |
| Nails only | 34.7 |
| Duration + PASI ≥10 | 32.9 |
| Nails + PASI ≥10 | 47.3 |
| Nails + Duration | 53.7 |
| High clinical risk | 62.6 |

Female. Waist circumference = 110 cm

| **Clinical scenario** | **Predicted probability (%)** |
| --- | --- |
| Low clinical risk | 14.9 |
| PASI ≥10 only | 20.9 |
| Duration ≥10 years only | 32.1 |
| Nails only | 42.5 |
| Duration + PASI ≥10 | 40.4 |
| Nails + PASI ≥10 | 55.6 |
| Nails + Duration | 61.9 |
| High clinical risk | 70.1 |

Male. Waist circumference = 90 cm

| **Clinical scenario** | **Predicted probability (%)** |
| --- | --- |
| Low clinical risk | 3.4 |
| PASI ≥10 only | 5.1 |
| Duration ≥10 years only | 9.4 |
| Nails only | 13.9 |
| Duration + PASI ≥10 | 12.7 |
| Nails + PASI ≥10 | 19.8 |
| Nails + Duration | 24.8 |
| High clinical risk | 33.2 |

Male. Waist circumference = 100 cm

| **Clinical scenario** | **Predicted probability (%)** |
| --- | --- |
| Low clinical risk | 4.6 |
| PASI ≥10 only | 6.8 |
| Duration ≥10 years only | 12.3 |
| Nails only | 18.3 |
| Duration + PASI ≥10 | 16.7 |
| Nails + PASI ≥10 | 25.6 |
| Nails + Duration | 31.5 |
| High clinical risk | 40.7 |

Male. Waist circumference = 110 cm

| **Clinical scenario** | **Predicted probability (%)** |
| --- | --- |
| Low clinical risk | 6.1 |
| PASI ≥10 only | 9.0 |
| Duration ≥10 years only | 16.0 |
| Nails only | 23.7 |
| Duration + PASI ≥10 | 21.6 |
| Nails + PASI ≥10 | 32.3 |
| Nails + Duration | 38.9 |
| High clinical risk | 49.3 |
